# Camouflaging moving objects: crypsis and masquerade

**DOI:** 10.1093/beheco/arx085

**Published:** 2017-06-17

**Authors:** Joanna R Hall, Roland Baddeley, Nicholas E Scott-Samuel, Adam J Shohet, Innes C Cuthill

**Affiliations:** a School of Experimental Psychology, University of Bristol, 12a Priory Road, Bristol BS8 1TU, UK,; b School of Biological Sciences, University of Bristol, Bristol Life Sciences Building, 24 Tyndall Avenue, Bristol BS8 1TQ, UK, and; c Stealth Materials Group, QinetiQ, Cody Technology Park, Farnborough GU14 0LX, UK

## Abstract

Motion is generally assumed to “break” camouflage. However, although camouflage cannot conceal a group of moving animals, it may impair a predator’s ability to single one out for attack, even if that discrimination is not based on a color difference. Here, we use a computer-based task in which humans had to detect the odd one out among moving objects, with “oddity” based on shape. All objects were either patterned or plain, and either matched the background or not. We show that there are advantages of matching both group-mates and the background. However, when patterned objects are on a plain background (i.e., no background matching), the advantage of being among similarly patterned distractors is only realized when the group size is larger (10 compared to 5). In a second experiment, we present a paradigm for testing how coloration interferes with target-distractor discrimination, based on an adaptive staircase procedure for establishing the threshold. We show that when the predator only has a short time for decision-making, displaying a similar pattern to the distractors and the background affords protection even when the difference in shape between target and distractors is large. We conclude that, even though motion breaks camouflage, being camouflaged could help group-living animals reduce the risk of being singled out for attack by predators.

## Lay Summary

We show that for objects moving in groups, spotting one that is a different shape is harder when the objects are similarly patterned. The difficulty of spotting the odd-one-out is further enhanced by matching the background and being in larger groups. So, even though motion ‘breaks’ camouflage, being camouflaged can help group-living animals reduce the risk of being singled out for attack by predators.

## INTRODUCTION

Camouflage of moving objects, particularly in terms of preventing detection, has often been assumed to be impossible (Regan and Beverley 1984; Ioannou and Krause 2009; Lui et al. 2012; Yin et al. 2015). As a result, the protection afforded moving objects by camouflage patterns has generally been under-researched (but see Zylinski et al. 2009; Josef et al. 2015) although the effect of conspicuous “dazzle” patterns has received more attention (Stevens et al. 2011; Scott-Samuel et al. 2011; Hughes et al. 2014; Hughes et al. 2015; Hall et al. 2016). However, combined evidence from visual search (e.g., Prinzmetal and Banks 1977; Farmer and Taylor 1980; Duncan and Humphreys 1989; Wolfe et al. 2002; Neider and Zelinsky 2006) and confusion effect studies (e.g., Krakauer 1995; Krause and Ruxton 2002; Ioannou et al. 2008; Scott-Samuel et al. 2015; Hogan, Cuthill, et al. 2016; Hogan, Scott-Samuel, et al. 2016; Hogan, Hildenbrandt, et al. 2017; Hogan, Cuthill, et al. 2017) suggests that targets moving on a complex background, surrounded by multiple similar objects (known in the visual search literature as distractors), could gain an advantage from a camouflage-type pattern, when compared to plain targets. Previous work in this area by Hall et al. (2013) provided evidence that camouflage patterning can slow the identification of a moving target when that target is displayed alongside multiple moving distractors that are similarly camouflaged, compared to when the target and distractors are plain gray. The type of camouflage pattern (background matching or disruptive) was found to be unimportant when the target was in motion. The authors suggested that the slowing of target identification is the result of the camouflage patterns enhancing the confusion effect.

Camouflage strategies are often considered in isolation. However, this is neither a requirement nor a realistic interpretation of the real world (see particularly Stevens et al. 2011). Multiple camouflage strategies could be exploited simultaneously to increase antipredation benefits, particularly in circumstances where the prey is at greater risk of detection, such as during movement. Under these circumstances, the benefits provided by each strategy may differ, and the impact of each, plus the overall effect of the combined strategies, can then be characterized. In the current experiments, “camouflage” encompasses both benefits from being a similar pattern to the background and from resembling irrelevant objects. The latter, usually termed masquerade, is distinguished from background matching because mimicry of irrelevant objects reduces predation risk even when viewed on nonmatching backgrounds (Skelhorn et al. 2011; Skelhorn et al. 2010a,b; Skelhorn and Ruxton 2011a,b). That is, masquerade affords camouflage through misidentification rather than concealment (Skelhorn et al. 2010c). Target-distractor similarity may therefore share some similarities with the mechanism(s) of masquerade, while target (and distractor) similarity to the background maps onto crypsis. These experiments, therefore, allow simultaneous investigations of multiple mechanisms in the context of moving prey.

It may seem counterintuitive to treat mutual similarity in a group of prey as akin to masquerade because all are edible, legitimate targets for attack. However, once the predator has singled out one individual as a target, which is frequently based on a difference in size, appearance or behavior (Krause and Ruxton 2002), then the other individuals in the group become potential distractors. If the difference between target and distractors is large, then there is an “oddity effect” and no confusion (Krakauer 1995; Ruxton et al. 2007; Tosh et al. 2009). However, if the target and nontarget prey are hard to discriminate then the target gains an advantage from its similarity to nontargets, just as in masquerade. Our experiments model this situation: where a target within a group must be selected based on a slight difference in phenotype (which we control experimentally). That difference is unrelated to coloration, but we then investigate whether being patterned, and being patterned like the background (i.e., camouflaged), affect the discrimination. The phenotypic attribute allowing discrimination is, for comparison with Hall et al. (2013), shape, but in principle we could have chosen any noncolor attribute, such as size or movement pattern. Our first experiment investigates whether target discrimination differs when faced with a patterned group on a plain background versus a plain group on a patterned background or a patterned group on a patterned background. We predict the latter should be the hardest context, as there is background matching in addition to target-distractor similarity. We also manipulate group size, which we predict should enhance difficulty for the predator. The experiment is replicated to see what effects persist when discrimination is made easier. A second experiment uses a different approach, based on an adaptive procedure to determine the threshold for discrimination based on target-distractor shape-similarity. This allows us to determine the magnitude of difference (between target and distractors) for which camouflage affords protection.

## GENERAL METHODS

All participants were naive to the purpose of the experiments, had normal or corrected-to-normal vision, and gave informed consent in accordance with the Declaration of Helsinki. The study was approved by the Research Ethics Committee of the Faculty of Science, University of Bristol. All trials were presented on a linearized (gamma-corrected), 22”, 1024 × 768 pixel laCie Electron 22Blue CRT monitor (LaCie Ltd., London) with a refresh rate of 100 Hz and a mean luminance of 21.7 cdm^−2^.

Backgrounds were static in all experiments. Patterns were generated from a first-order autoregressive spatial process with normal error distribution (Yearsley 2004), producing patterns with a coarse, local spatial structure that bore some of the fractal properties of natural backgrounds (and modern military camouflage; for details of pattern generation see Hall et al. 2013, Supplementary Methods). The mean RGB values of the patterned backgrounds were 91 and the pixel values had a range of 0–255. Plain backgrounds and targets matched the mean luminance of the patterned background. New distractors, background, and target were generated for each trial.

## EXPERIMENT 1A,B: BACKGROUND MATCHING AND TARGET-DISTRACTOR SIMILARITY

### Background

In Hall et al. (2013), a target was detected among distractors on the basis of shape (an elliptical target amongst circular distractors). In any one treatment, these objects were either all plain or all patterned, against a background pattern that was similar to those on the patterned objects. These conditions can therefore be considered as target-distractor discrimination in a context in which the targets either have background-matching camouflage, or not. However, in this previous work, similarity to the background is coincident with patterning of the targets per se, so a third condition—patterned, but not matching the background—is required in order to evaluate the relative importance of displaying a pattern and hiding on a patterned background. The fourth condition of a 2 × 2 design, plain targets on a plain background, is not possible because background matching is perfect (indeed to the viewer, if not the computer programmer, targets do not exist).

Targets were ellipses and the distractors circles. The experiment was replicated twice with different magnitudes of differences between targets and distractors: in experiment 1a, the ellipsoid targets had a minor axis 0.7 times that of the major axis; in experiment 1b, the ratio was 0.6, a slightly easier discrimination. Although trials where distractor patterns are very dissimilar to the target pattern have previously been shown to be equivalent to trials with no distractors (Hall et al. 2013), it is of interest to determine whether any effects of pattern are evident at different levels of discrimination difficulty. Distractor number was also varied, with either 5 or 10 distractors present in each trial. Hall et al. (2013) reported that target detection was slower with 10 distractors than 5 and the same result was predicted for the current experiment.

### Methods

In experiment 1a, elliptical targets (area: 2400 pixels; minor axis = 0.7 × major axis; major axis: 1.6 deg; minor axis: 1.1 deg) and circular distractors (diameter: 1.4 deg, same area as ellipse) were generated in 8 different patterns (plain, background matching, step edge, step centre, graduating edge, graduating centre, disruptive edge, and disruptive centre; see Supplementary Material), exactly as in Hall et al. (2013). The target and distractors moved at 4 degrees per second in any direction with no occlusion, rebounding off each other as well as the boundary at an angle equal to the angle of incidence.

Each subexperiment comprised 600 trials, split between 2 blocks based on the background. The patterned background block consisted of 320 trials (8 target patterns × 2 distractor numbers × 20 replicates) and the plain background block consisted of 280 trials (dropping the Plain treatment for reasons discussed above: 7 target patterns x 2 distractor numbers × 20 replicates). Presentation of the blocks was counterbalanced and trials within each block were presented in random order. In each trial, 5 or 10 circular distractors were displayed on screen, alongside the elliptical target, which had its major axis randomly oriented either vertically or horizontally. The distractors and target always displayed the same type of pattern as each other. However, within any one pattern type, all targets and distractors, within and across all trials, were unique random generations of that pattern type (e.g., for “background matching” patterns, every ellipse or circle was different).

In each trial, participants had to identify the elliptical target and indicate its orientation. Identification of the target was not measured with a mouse click on the target, as this would be a joint product of identification and motor skill in target tracking. The latter is undoubtedly an important determinant of predation success in many systems, and may be affected by prey coloration (Hogan, Cuthill, et al. 2016), but our objective was to isolate the effect of coloration on identification. So, to measure this, participants were asked to indicate the orientation of the elliptical target (horizontal or vertical) as quickly and accurately as possible with a key press. Each trial continued until the participant responded. Response times and accuracy of responses were recorded. Participants, naive to the object of the experiment, were recruited from the undergraduate population at the University of Bristol and completed the experiment for course credits. Inclusion criteria were set prior to the experiment so that only participants who scored a minimum of 90% correct on both blocks would be included in the analysis. This allowed the assumption that response times were not influenced by guessing, that is, the result of a speed-accuracy trade-off. Eleven participants were tested to find 10 that met this criterion; analysis was then carried out on all correct trials.

**Figure 1:**
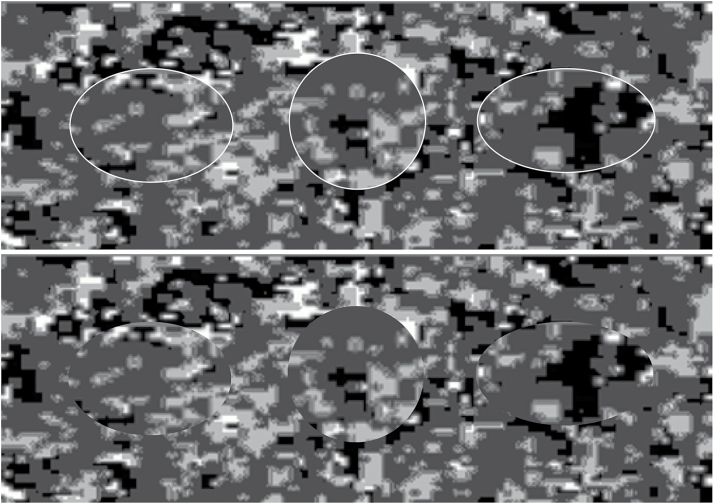
Comparison of target and distractor shapes in experiment 1. Top panel shows objects with white outlines to illustrate shape differences (white outlines were not part of experimental stimuli), bottom panel shows same objects without white outlines. Left: ellipse with minor axis = 0.7 × major axis (experiment 1a), middle: circular distractor (minor axis = major axis; used in both experiments), and right: ellipse with minor axis = 0.6 × major axis (experiment 1b).

In experiment 1b, the similarity between target and distractors was decreased by increasing the eccentricity of the elliptical target. The surface area of the target remained the same as that of the distractors (2400 pixels). The eccentricity was increased so that the minor axis was 0.6 times that of the major axis (major axis: 1.7 deg; minor axis: 1.0 deg; see Figure 1 for comparison to distractor and previous target shape). All other aspects of the experiment remained the same as in 1a. Another 10 undergraduates, also naive to the object of the experiment, from Experimental Psychology at University of Bristol were recruited and completed the experiment for course credits. All participants achieved the minimum of 90% correct in each block and were included in the final analysis.

Data for each experiment were analyzed with a repeated-measures model ANOVA (with pattern-background combination and distractor number as within-subjects factors and participant as a random effect) implemented with function aov in the R environment (R Core Team 2015). Post hoc tests for pair-wise comparison of pattern-background combinations (3 levels) were carried out using linear contrasts with *P*-values adjusted, with function *P*adjust, to control the false discovery rate (Benjamini and Hochberg 1995).

### Results

As in Hall et al. (2013) for moving targets, the specific pattern had no effect on response times (see Supplementary Material) and so the results for the pattern subtypes are pooled in this experiment.

**Figure 2:**
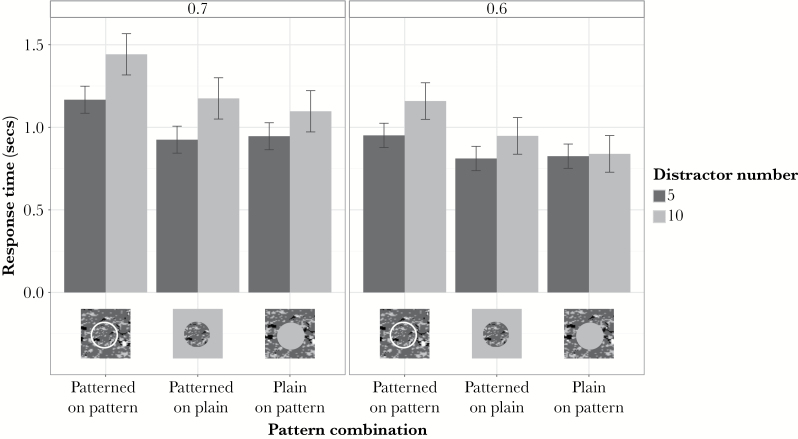
Mean response times (± SEM) for identification of target with a major to minor axis ratio of 0.7 (left panel) and 0.6 (right panel) for different pattern combinations and 5 or 10 distractors.

In experiment 1a (Figure 2, left panel), response times for identifying the orientation of the elliptical target were affected by target-background pattern combination (F_2,18_ = 30.94, *P* < 0.0001) and the number of distractors present (F_1,9_ = 137.40, *P* < 0.0001) but the interaction was not significant (F_1,9_ = 3.53, *P* = 0.0508). Responses were, on average, 0.22 s slower when there were more distractors present (ca. 20%). Patterned targets on patterned backgrounds produced longer response times than patterned targets on plain backgrounds (mean difference 0.25 s; *P* < 0.0001) or plain targets on patterned backgrounds (mean difference 0.28 s; *P* < 0.0001). Plain on patterned and patterned on plain had, however, similar response times (mean difference 0.03 s; *P* = 0.4811). Given the nearness of the interaction to significance, it would be negligent to assume a null effect. So, we also analyzed the effect of target-background pattern combination separately for 5 and 10 distractor conditions. For 5 distractors, pattern had an effect (F_2,18_ = 27.53, *P* < 0.0001) with the treatment differences similar to the combined results presented above. Patterned on patterned had longer RTs than patterned on plain (mean difference 0.24 s; *P* < 0.0001) or plain on patterned (mean difference 0.22 s; *P* < 0.0001), with patterned on plain similar to plain on patterned (mean difference 0.02 s; *P* = 0.5683). For 10 distractors, pattern also had an effect (F_2,18_ = 19.04, *P* < 0.0001) with the treatment differences also similar to the above. Patterned on patterned had longer RTs than patterned on plain (mean difference 0.26 s; *P* = 0.0005) or plain on patterned (mean difference 0.34 s; *P* < 0.0001), with patterned on plain nonsignificantly longer than plain on patterned (mean difference 0.08 s; *P* = 0.1750).

The effects of distractor number and pattern are similar across experiments 1a and 1b (detailed results of experiment 1b can be found in the Supplementary Material). There was a significant interaction (*P* < 0.001) between distractor number and pattern in 1b (Figure 2, right panel), noteworthy given the near-significant interaction in 1a. The interaction arose because, with 10 distractors, there is a tendency toward identification taking slightly longer for patterned on plain than the reverse, while with 5 distractors the means are very similar (Figure 2). Nevertheless, regardless of distractor number, the RTs for patterned on patterned are consistently longer than for patterned on plain or plain on patterned.

## EXPERIMENT 2: TARGET-DISTRACTOR SIMILARITY AS A TOOL FOR TESTING CAMOUFLAGE STRATEGIES

### Background

While most previous literature has concentrated on camouflage when targets are stationary, more recent publications (Stevens et al. 2011; Hall et al. 2013; Hughes et al. 2014, 2015; Hogan, Cuthill, et al. 2016; Hogan, Scott-Samuel, et al. 2016) have investigated the effects of camouflage for moving targets. However, most situations in the real world are likely to combine these 2 eventualities: For example, prey animals will move freely unless they detect the presence of a predator, at which point they become stationary and wait for the predator to move off. It therefore follows that camouflage need only provide an advantage during motion for the short duration before the prey detects the presence of a predator and becomes motionless, at which point an effective camouflage pattern can render the prey almost undetectable. Previous experiments have shown that matching the pattern of backgrounds and distractors can slow identification of a moving target (Hall et al. 2013) and, combined with short observations, this may be all that is required to reduce the risk of predation. The effect will increase further if the predator is not specifically aware of the presence of the prey. In this case the predator would only perceive a moving object, with its identity obscured, for a short duration and then no further information would be available unless it is able to discover the stationary prey. Other examples in which camouflage could be useful for short durations include situations where prey movement only occurs when the animal moves between patches, so the camouflage only need provide an advantage for the short duration in which the animal changes patch, or when predators do not pay attention to one spot, but broadly inspect a whole scene by moving their visual focus. In this case, camouflage can provide an advantage as long as it is effective for longer than the predator spends attending to any one spot.

In the first 2 experiments, a greater difference in shape between the target and distractors resulted in quicker response times. If this difference between the target and distractor shape were taken to the extreme, an oddity effect would occur, reducing the confusion effect by increasing targeting accuracy (Krakauer 1995; Ruxton et al. 2007). However, if camouflage patterns can slow shape recognition, it follows that more effective camouflage strategies should tolerate a greater difference in shape between target and distractors before they fail. Therefore, it should be possible to exploit target-distractor shape similarity as a tool to probe the effectiveness of different camouflage strategies. In this experiment, we test the simple comparison of being patterned on a matching patterned background versus being patterned on a plain background. However, other camouflage strategies, including different patterns types (e.g., organic versus geometric patterns, stripes versus zigzags), color change or even movement patterns, could be tested in future.

In order to investigate this potential tool, we established the maximum similarity in shape between target and distractors, under different conditions, at which the target could still be differentiated from the other objects. In essence, we defined the threshold, for each of the conditions, at which identification of the target was no longer reliable. “Staircase” or “adaptive” methods (Cornsweet 1962) provide a useful method for establishing thresholds for many psychological parameters (e.g., Palmer 1995; Palmer et al. 2000; Seiple et al. 2001). When participants find discrimination easy, it is made more difficult, and vice versa, until a point of equilibrium is reached which defines the threshold for discrimination. In this experiment, the ratio of major to minor axis was manipulated between trials, according to participant accuracy, to gradually increase or decrease the similarity of the target shapes to that of the circular distractors. This provided the opportunity to investigate the benefits of camouflage using a different approach from that in the previous experiments. Two backgrounds (plain and patterned) and 2 display durations (short and long) were tested in a 2 × 2 design and the threshold for identifying the patterned ellipse was established for the 4 conditions. The short duration, background matching condition was expected to result in a threshold with the greatest difference between target and distractors, and vice versa for the long duration, nonbackground matching condition.

### Methods

This experiment used elliptical targets and circular distractors as in the previous experiment but, in this case, both the background pattern and the display duration were manipulated. This resulted in a 2-backgrounds (plain or patterned) × 2-display duration (short: 200 ms, long 1000 ms) design. A pilot study was used to identify a short duration that still allowed the task to be completed by naive participants.

As experiments 1a and b (and Hall et al. 2013) had shown no difference between the specific camouflage patterns used, the design was simplified so that all objects in this experiment displayed the background-matching pattern. The background-matching targets and distractors and the backgrounds were produced in the same manner as those in experiment 1.

The experiment aimed to find the threshold at which discrimination of the target was no longer possible, based on the difference in shape between the target and distractors, for the 4 conditions (short viewing duration, matching background; long viewing duration, matching background; short duration, not matching background; and long duration, not matching background). The difference in shape between the target and distractors was manipulated by controlling the eccentricity of the ellipse. This was measured simply by the ratio of the major axis to the minor axis: a ratio of 1 would produce a circle, and a ratio of 0.1 would produce a highly elongated ellipse. For the experiment, the ellipse ratio could range from 0.4 to 0.95 in steps of 0.025. The surface area of the ellipse was constant at 2400 pixels, the same as for the circular distractors (1.4 deg diameter).

In the first trial for each condition, the ellipse was presented with a major:minor axis ratio of 0.5. The eccentricity of the ellipse then followed a two up, one down staircase: if the participant was correct twice the ratio increased a step, making the ellipse more circular and the task harder. However, if the participant answered incorrectly at any point, the ratio reduced by a step. The experiment was designed so that if the ratio reduced to the minimum of 0.4 and the participant still answered incorrectly the trials would repeat at this ratio until the participant answered correctly twice in a row. However, no participants answered incorrectly when the ellipse was this elongated. Similarly, the task would just repeat the maximum ratio of 0.95 if this were reached; however, this did not happen either. This staircase method allowed the threshold for detecting the target to be tracked for each condition individually. In order to avoid any effects on the staircases resulting from different ellipse orientations, for example, if participants always use the horizontal axis to compare to the distractor diameter regardless of whether this is the longer or shorter axis, we used a target present/absent design rather than asking participants to identify the orientation of the target as in experiment 1. It was therefore necessary to ensure that participants could not tell whether there would be an ellipse present in each trial. In order to achieve this, half of the trials contained no ellipse. The responses from these trials were not used to calculate the staircases but ensured that the participants were completing the experiment in the required manner.

Each trial consisted of 5 patterned objects displayed on a background square for the required duration. The objects again all moved at 4 deg/s with ballistic motion, rebounding off each other and the boundaries. In trials where no target was present, all 5 objects were circular distractors. In trials where the target was present, the objects consisted of one ellipse and 4 circular distractors. The ellipse was always displayed with its major axis in a horizontal orientation. After the objects had been displayed for the required duration, another complex pattern (created in the same way as the background) was displayed on the screen as a mask and the participant was asked to indicate whether they had observed an elliptical target via a key press: using “s” if there was a target present and “k” if there was no target present. Participants were informed at the start of the experiment that response times were not important.

In total, the experiment consisted of 512 trials: 256 contained no target and the other 256 consisted of 64 trials for each of the 4 conditions. Trials for the different conditions were mixed at random and the experiment was split into 5 blocks, each of just over 100 trials, to provide the participants with the opportunity to take breaks in order to maintain their concentration.

For each participant the mean difference between target and distractor aspect ratios was calculated for the last 4 reversals for each condition, following Seiple et al. (2001). The thresholds for target present trials were then analyzed using a repeated measures ANOVA, implemented via the aov function in R.

Ten postgraduate students, naive to the object of the experiment, were recruited from the School of Experimental Psychology, University of Bristol and were reimbursed for their time.

### Results

The mean accuracy for target absent trials was high in all conditions (short duration, matching the background: 86.7%; long duration, matching the background: 97.5%; short, not matching the background: 86.4%; long, not matching the background: 98.4%), showing that participants were completing the experiment in the required manner. Analysis showed a significant effect of duration on accuracy in the target absent trials but no effect of matching the background, nor a significant interaction between the 2 factors (main effect of duration F_1,9_ = 15.22, *P* = 0.004; main effect of match F_1,9_ = 0.112, *P* = 0.735; interaction F_1,32_ = 0.474, *P* = 0.509).

**Figure 3:**
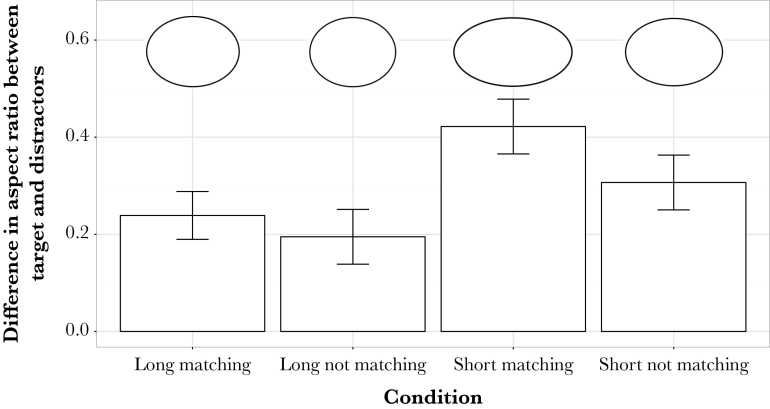
Mean thresholds (± SEM) for the 4 conditions, after being averaged over the last 4 reversals and over all participants. The short, matching condition has the highest threshold meaning that for this condition the camouflage is effective for the greatest difference between target and distractors.

For target present trials, the conditions with long presentation durations produced lower thresholds than the conditions with short durations (F_1,9_ = 45.57, *P* < 0.001) and so too did the background matching conditions compared to the nonmatching conditions (F_1,9_ = 30.97, *P* < 0.001). The interaction between the two was not significant (F_1,9_ = 4.997, *P* = 0.052), with the trend being for a greater effect of matching the background for short than long durations (Figure 3). The short duration, background-matching condition produced the highest threshold; for this condition the camouflage was effective for the greatest difference in shape between target and distractors (i.e., when the target was least similar to the distractors: an aspect ratio of ca 0.58 vs. 1.0). The long duration, not background-matching condition produced the lowest threshold, where the target was closest in shape to the distractors (an aspect ratio of ca 0.8 vs. 1.0). The key result is that matching the pattern of the background allows targets to be less similar in shape to the distractors and still gain protection from the camouflage, compared to when they do not match the background.

## DISCUSSION

Before discussing the results it is useful to be clear about the sort of real-world situation to which these results might apply. First, we have modeled a situation in which a target must be discriminated from distractors based on a shape difference. We chose shape for comparison with Hall et al. (2013) but, for predators attacking natural prey groups, other cues for separating a target may be more important: proximity, location (temporary separation from others), size, relative speed or trajectory, or other behavioral differences (Landeau and Terborgh 1986; Tosh et al. 2006). We make no special claims for shape as a discriminatory stimulus but it is an empirical question whether coloration interferes with other cues to the same degree. The second issue in applying our results to the real world is the nature of the distractors. We conceived the experiment as applying to a moving group of animals, where the distractors are other potential, but unselected, prey. However, the experiments also may correspond to a situation where the distractors are irrelevant, inedible, objects and here the protection is akin to masquerade. It is perhaps harder to think of situations where a prey item is surrounded by similarly colored, similarly moving, nonprey objects, but a planktonic animal in moving water where there are suspended particles may correspond to this, particularly if the predator has poor visual resolution.

In experiment 1, the patterned objects displayed on a patterned background resulted in the slowest identification. Consistent with the result of our previous study (Hall et al. 2013), the benefit of background matching was enhanced when the number of distractors was increased, presumably due to the confusion effect (Ioannou et al. 2008; Krakauer 1995; Krause and Ruxton 2002; Ioannou et al. 2008; Scott-Samuel et al. 2015; Hogan, Cuthill, et al. 2016; Hogan, Scott-Samuel, et al. 2016). The identity of a target is best concealed when it matches the pattern of both the background and distractors. We found some evidence of discrimination being slower for a patterned group on a plain background than for a plain group on a patterned background but only for 10 distractors. A plausible reason for this is that, although segmentation from the background is easy in both cases, when targets and distractors are plain, participants can concentrate on the only difference, shape. However, when targets and distractors are patterned, because each pattern was uniquely generated (with same algorithm but a random seed), there are small target-distractor and distractor-distractor differences that might divert attention somewhat from the shape discrimination task. The response times for both of these conditions are still longer than those previously reported for trials where no distractors were present (Hall et al. 2013). Some enhancement of the confusion effect was also seen when target shape was more similar to distractor shape (RTs were 22% longer, on average, in experiment 1a than 1b: 1.13 vs. 0.92 s) but these were different experiments. We would need to manipulate aspect ratio within a single experiment to be sure that shape similarity was the cause of the observed differences. The more important point is that background matching and distractor number had consistent effects in both experiments.

The patterned background in this experiment matched the pattern on the targets but also contained a high degree of complexity. Increased background complexity has previously been shown to increase the time for stationary target detection in humans and birds (e.g., Wolfe et al. 2002; Neider and Zelinsky 2006; Dimitrova and Merilaita 2010; Xiao and Cuthill 2016), while killifish have been shown to display differences in their preferences for complex backgrounds versus pattern-matching backgrounds (Kjernsmo and Merilaita 2012). Dimitrova and Merilaita (2012) reported that the risk of a stationary target being detected is affected by a complex relationship between prey pattern and background complexity. Further investigations will therefore be required to establish how background matching and background complexity interact when objects are in motion.

In experiment 2, as predicted, there was an effect of matching the background pattern, with thresholds for the matching conditions being higher than in the conditions where the objects were patterned and the background was plain. When the objects matched the background pattern, the target was difficult to identify even when its shape was different from that of the distractors. However, when the background was plain, a target with a similar aspect ratio to the distractors was still easy to discriminate. This is consistent with results from the previous experiments, showing that background pattern plays an important role in camouflaging moving objects.

For the short duration condition where the objects matched the background pattern, the threshold occurred at the highest difference in aspect ratio (when the minor axis was less than 60% the length of the major axis for the target, compared to 100% in circular distractors). The long duration, nonmatching condition produced the lowest difference threshold (minor axis over 80% the length of the major axis). So, it is generally better to be observed for the shortest possible amount of time or to match the background. However, we cannot rule out the possibility that a prey may gain a further advantage in hiding its identity if the target is able to satisfy both of these criteria; the interaction was not significant at *P* = 0.052. Under these conditions, camouflage can still reduce identification, even when the shape of the target differs quite markedly from that of nontarget individuals.

Shorter duration conditions produced lower thresholds irrespective of whether the objects matched the background, although this is not surprising. The mean duration for a single fixation in humans performing visual search is 180–275 ms (Rayner 2009) and the short duration in this experiment was 200 ms; thus in this condition, the participants had only a single fixation to identify whether the target was present. The longer duration lasted 1000 ms and therefore provided time for participants to inspect, that is, foveate, the objects with multiple fixations. So, the 2 durations were quantitatively but also qualitatively different. This qualitative difference between a single fixation and closer inspection appears to have a greater effect on the performance of the camouflage than the difference between matching and not matching the background.

With such a controlled experimental set up, there are necessarily limitations when generalizing to the wider world. For example, shape is not the only feature available for predators to exploit for prey choice and there is evidence that shape is not the most salient cue used by predators (e.g., Kazemi et al. 2014; Sherratt et al. 2015). However, it should be noted that disruptive coloration is hypothesized to be a camouflage mechanism that specifically allows an animal to not only match the background but also to break up it’s body outline in order to reduce shape recognition, so it is likely that predators do attend to object shape in at least some situations. Another issue is that in the natural world, differences in shape may correlate with other factors: For example, juveniles may be a different shape to adults but they may also differ in factors such as size and speed of movement which could impact on various stages of the predation process which we have not modeled in this particular study. In our study, the participants were told which object to target rather than being allowed to make their own choice, again slightly reducing the ecological validity of the system but this could prove to be an interesting avenue for further research into the impacts of coloration on predator target selection and capture performance. While our study did not address this directly, the results are consistent with those from multiple object tracking studies. This related area provides evidence that when objects are very similar in appearance, tracking performance is impeded (e.g., Feria 2012; Howe and Holcombe 2012). Whilst we did not investigate tracking in our study, it is the next stage in the predation process and it is therefore pertinent that the strategy of visually matching other nearby objects, and the background where possible, can provide an advantage not just by reducing object identification but also by disrupting object tracking.

The staircase method used in this experiment, being an adaptive procedure, shares similarities with in silico selection experiments, where prey characteristics evolve in response to predator attack preferences (Bond and Kamil, 2002, 2006). These are a powerful approach for testing the success of different camouflage strategies. In our experiment, for example, it gives us the degree of prey oddity that can be concealed by a given color pattern, and thus provides a method to compare many other patterns or colorations in the future.

## CONCLUSIONS

Through these experiments, we have shown that both target-background and target-distractor pattern similarity combine to slow the process of identification in moving targets and that the benefits of these strategies can be enhanced by increasing the number of individuals in a herd or shoal. Masquerade is well known to provide benefits for stationary targets but here we have highlighted that benefits can also be gained from matching other nearby objects when in motion.

## SUPPLEMENTARY MATERIAL

Supplementary data are available at *Behavioral Ecology* online.

## FUNDING

This work was supported by a CASE Studentship to J.R.H. funded by the EPSRC (grant no. EP/C537556/1) and QinetiQ.

## Supplementary Material

Paper2_Supplementary_Material
