# Supplementary material for: Camouflaging moving objects: crypsis and masquerade
Source: Behav Ecol. 2017 Jun 17;28(5):1248–55. doi: 10.1093/beheco/arx085 (PMC5873248; doi:10.1093/beheco/arx085)
Supplement: Paper2_Supplementary_Material [file arx085_suppl_paper2_supplementary_material.doc]

**Supplementary Material**

**Camouflaging Moving Objects: Crypsis and Masquerade**

**Target Patterns**

Targets were generated in eight different patterns as illustrated in Figure 1 (figure and legend taken from Hall et al. 2013).


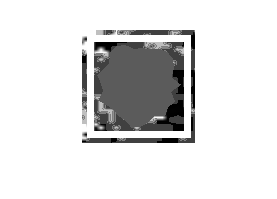

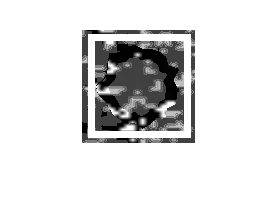

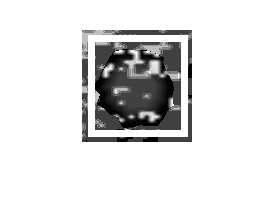

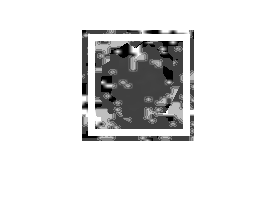


**b**

**d**

**f**

**h**

**a**

**c**

**e**

**g**


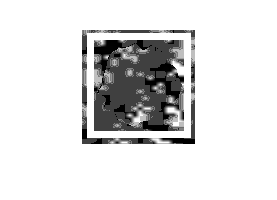

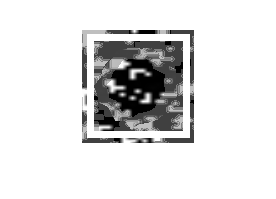

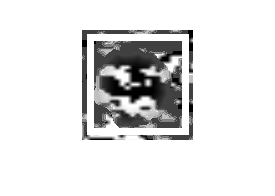

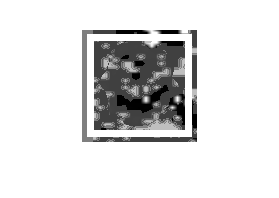


“Figure 1. Sample stimuli patterns. Artificial patterns were generated and used for stimuli: (a) no pattern (NP), (b) background matching (BM), (c) step edge (SE), (d ) step centre (SC), (e) graduating edge (GE), (f ) graduating centre (GC), (g) disruptive edge (DE) and (h) disruptive centre (DC). Each pattern implements a camouflage strategy previously proposed in the literature.”

**Generation of Backgrounds and Targets**

The following methods, describing generation of backgrounds and generation of target patterns, are quoted verbatim from Hall et al. (2013) Supplemental Methods (http://rspb.royalsocietypublishing.org/highwire/filestream/46766/field_highwire_adjunct_files/0/rspb20130064supp1.doc):

**“Backgrounds**

Artificial backgrounds were used, rather than real, in order to be able to generate multiple replicates with the same statistical properties. For each trial, a first-order autoregressive spatial process with normal error distribution (Yearsley 2004) was used to create two greyscale (luminance range: 0-255 in integer steps) patterns (pattern 1: autocorrelation = 0.2, variance of error = 1; pattern 2: autocorrelation = 0.235, variance of error = 1). This resulted in two patterns in which the majority of elements were very dark or very light (see Figure 1). In order to investigate the importance of maximum contrast for disruptive coloration, it was necessary to ensure that the background would contain some dark and light patches but also some lower contrast areas. Without the lower contrast areas, high contrast disruptive patterns used on targets would have appeared the same as the rest of the target and so there would have been little difference between the disruptive and background matching patterns. Therefore, the contrast of Pattern 1 was manipulated based on Pattern 2, in order to produce another greyscale pattern with a greater proportion of low contrast areas. This was achieved by finding each pixel in Pattern 2 with a luminance of less than 250. The corresponding pixels (in terms of position) in Pattern 1 were then checked and, if their luminance was less than 64 or greater than 191 (*i.e.* in the lower quarter or upper quarter of the luminance range), the value was adjusted towards mean luminance (127.5), in ratio with the original value. An example of the resulting pattern can be seen in Figure 2, Final background. Background patterns were used both for generating the target patterns and as the background in experimental trials. However, different sets of backgrounds were generated for each use.


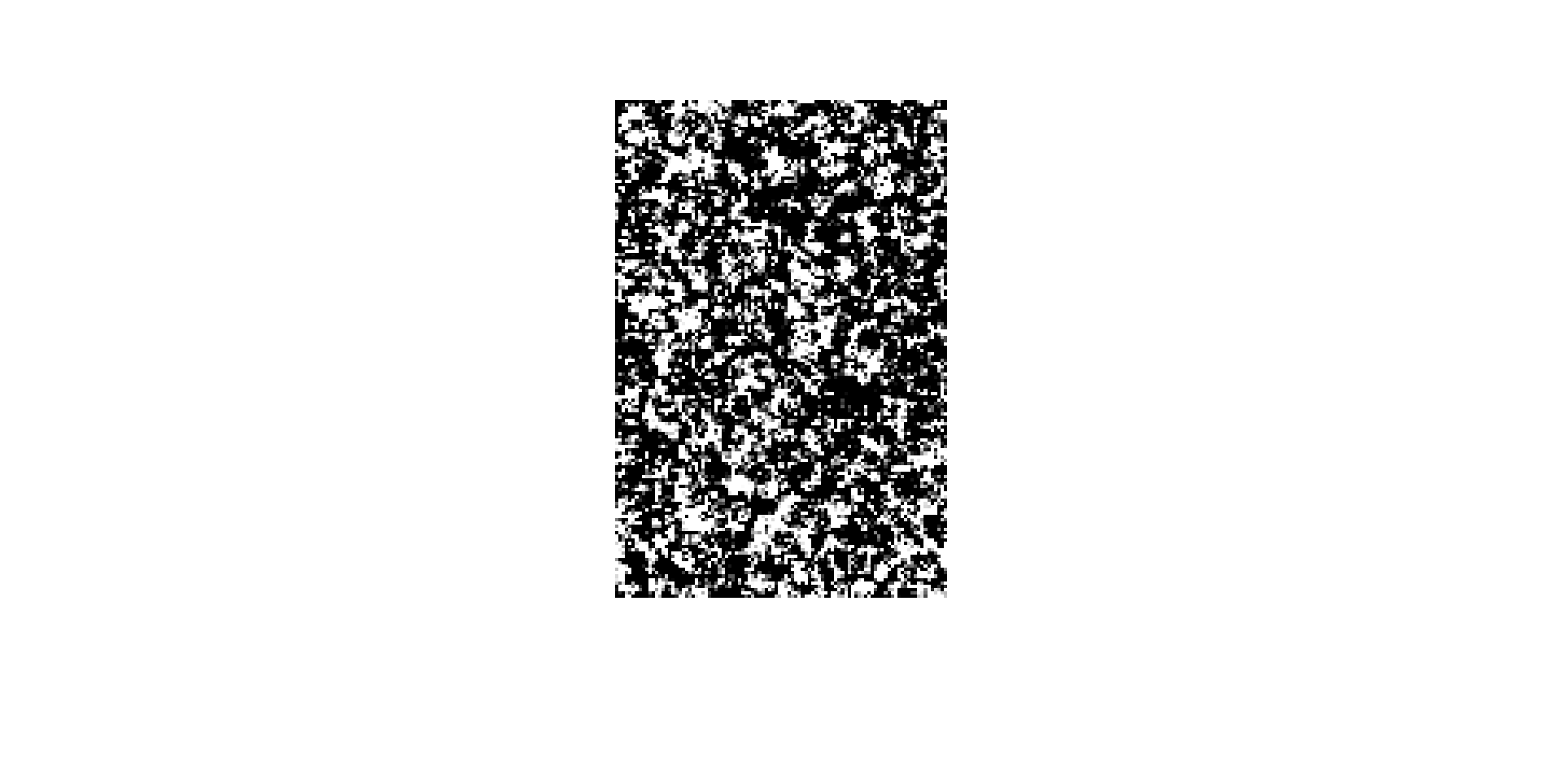

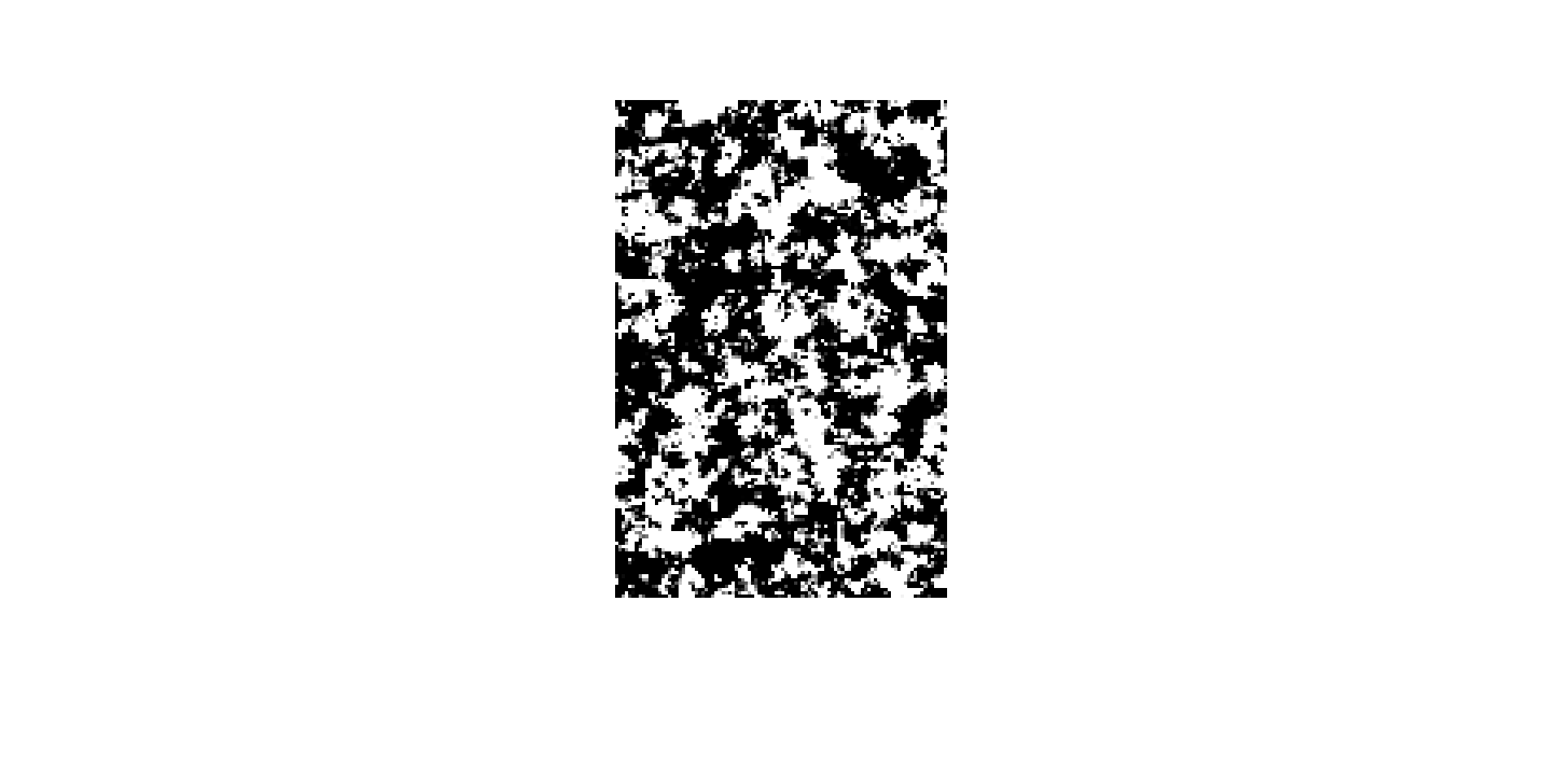

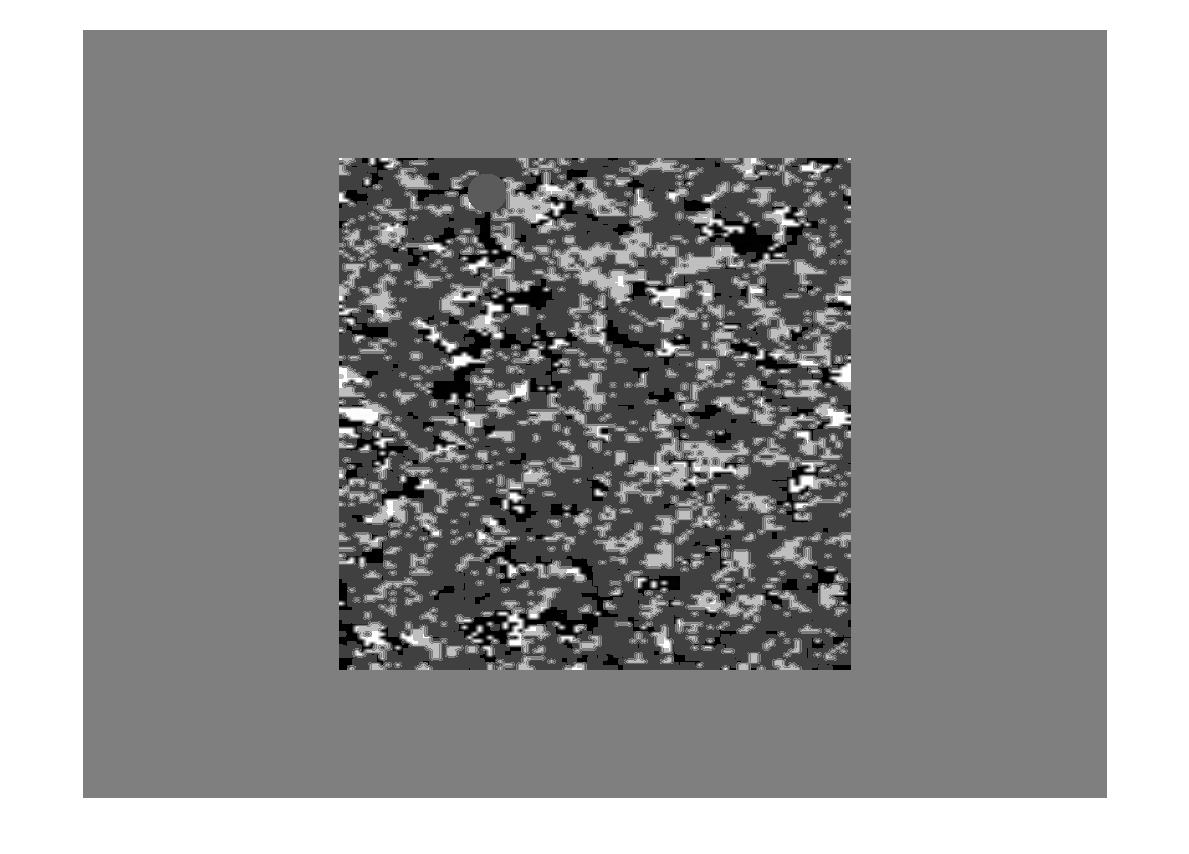


Pattern 1

Pattern 2

Final background

Figure 2. Samples of the patterns that were generated and the final background produced. Two patterns were generated with different spatial characteristics. Pattern 1 was then manipulated based on Pattern 2 in order to create the final background pattern for use in target generation and experimental trials.

**Target Patterns**

The target patterns were generated by sampling the background patterns and manipulating them in different ways. For every target generated, a new background pattern was created and the position of sampling was randomized.

*No pattern*: This target was monochrome with luminance of 91. This luminance is equal to the mean luminance of the background matching targets.

*Background matching*: A sample of the background with no further manipulations.

*Step edge*: In Pattern 1 (see Figure 2.) the lightest elements were white, (luminance 255) and the darkest elements were black (luminance 0), so the initial contrast was 100%. A sample of this pattern was taken and the contrast in the inner half of the surface area was reduced to 50% by manipulating the luminance so that all pixels fell in the luminance range 64-191. In order to do this any pixels with a luminance of less than 64 or greater than 191 (referred to in future as ‘luminance limits’) were identified and the luminance of the former was increased by 64 and the latter decreased by 64. The outer half of the target was left in its initial high contrast state (see Figure 3. *Left*).

*Step centre*: Generated in the same way as step edge but the outer half was manipulated, leaving the inner half as the high contrast area.

*Graduating edge:* A sample of Pattern 1 was taken. The outer quarter of the surface area was left unchanged to form the high contrast area. The inner quarter was manipulated in the same manner as the inner half in the step edge target (with luminance limits of 64 and 191) to produce the low contrast area. The remaining half of the surface area, between the high and low contrast areas was split into rings of 100 pixels in area. The total lower luminance limit (64) was then divided by the number of rings in the target to calculate the increase required for each graduation. For example, with 4 rings each graduation would be 64/4=16 more than the last. The luminance limits for each ring were then calculated by multiplying the graduation difference with the position of the ring. So continuing the 4 ring example, the inner of 4 rings would have a lower limit of 16x1 = 16, and the upper limit would be found by 255-(16x1) =239. The next ring would have a lower limit of 16x2 and an upper limit of 255-(16x2), and so on. The contrast in each ring was then manipulated in the same manner as in the step targets but taking into account each ring’s individual luminance limits, to give a graduation across the rings (see Figure 3. *Right*).

*Graduating centre*: Generated in the same way as graduating edge but this time the high contrast area was in the centre and the rings graduated in the opposite direction.

Figure 3. Diagram of target generation. Black denotes areas of high contrast and white denotes areas of low contrast. *Left*: Step edge, where contrast between pixels in the inner half of the surface area was reduced to 128 while contrast in the outer half remained at 256. *Right*: Graduating edge, where the high contrast area (256) was in the outside quarter and contrast graduated down to the low contrast area (128) in the inside quarter.

*Disruptive edge*: A third pattern was created from the first-order autoregressive spatial process with normal error distribution (Yearsley 2004), with autocorrelation = 0.239 and variance of error = 1. The spatial structures in this third pattern were larger than in the other patterns created, producing a suitable template for generating disruptive markings (see Figure 4). In the next stage, a sample was taken from Pattern 1 and the inner half of the surface area was manipulated as in the step edge targets to produce the low contrast area (luminance limits 64 and 191). For the high contrast area (the outer half) the Pattern 1 sample was compared to a sample of Pattern 3. For any pixels in the sample of Pattern 3 where the luminance was less than 80, the corresponding pixel in the Pattern 1 sample was checked. If its luminance was less than 64 or greater than 191, the value was adjusted towards mean luminance in ratio with the original value. This produced clumps of high contrast white and black pixels (disruptive markings) amongst lower contrast background matching areas.

*Disruptive centre*: Generated in the same way as disruptive edge but the high contrast patches were in the centre half.


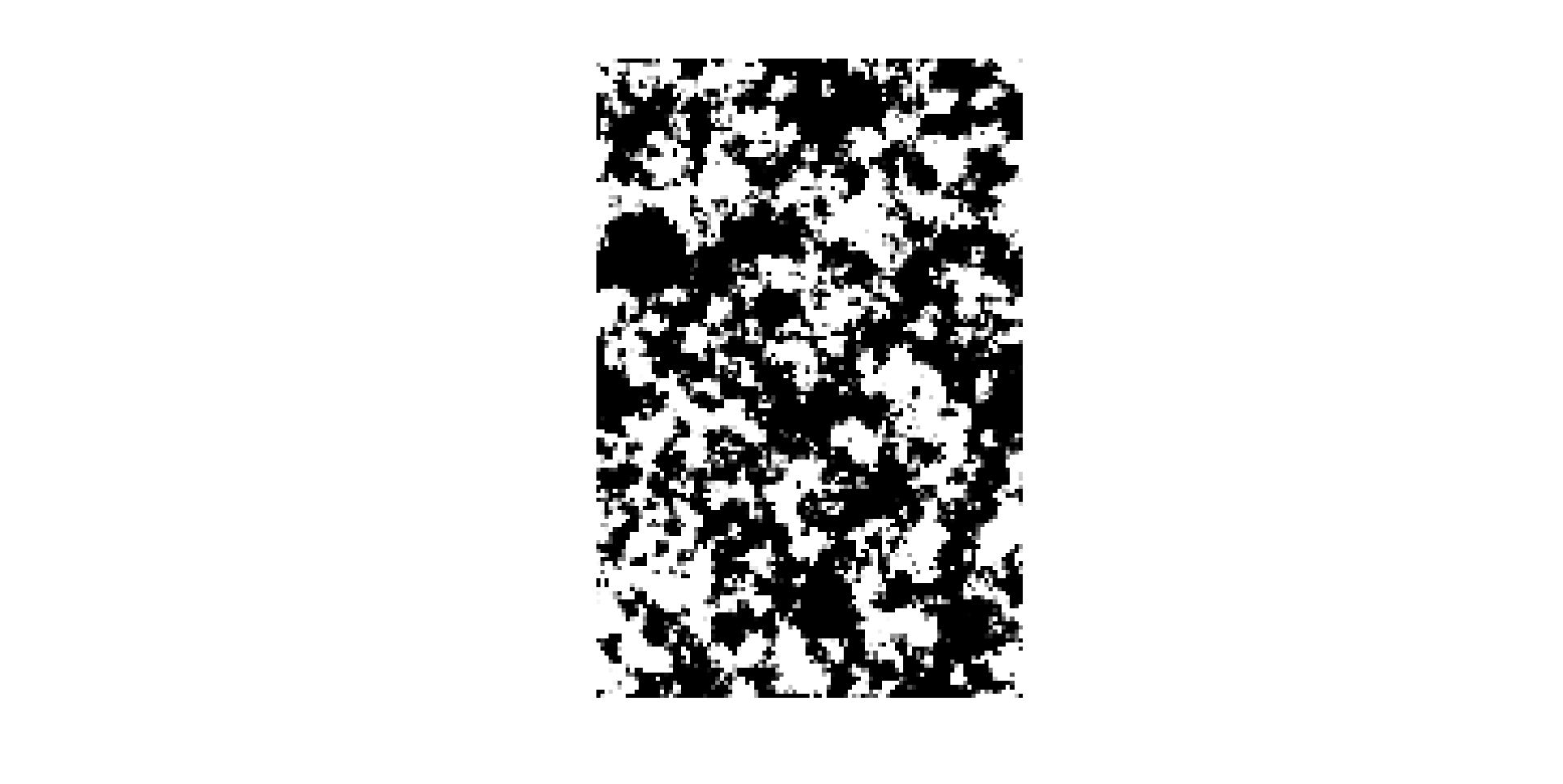


Figure 4. A third pattern with different spatial characteristics was generated in order to create the disruptive targets.”

**Experiments 1a and 1b: No effect of pattern sub-types on response times**

The effect of the different pattern sub-types (background matching, step edge, step center, graduating edge, graduating center, disruptive edge and disruptive center) on response times were compared using a repeated measures ANOVA and there was found to be no effect of pattern for Experiment 1a (F_6,266_ = 0.213, p = 0.972) or Experiment 1b (F_6,266_ = 0.165, p = 0.986). As a result the pattern sub-types were pooled prior to further analysis and comparison with the plain targets.

**Experiment 1b: Detailed results**

In experiment 1b response times for identifying the orientation of the elliptical target were affected by target-background pattern combination (F_2,18_ = 19.97, p < 0.0001) and the number of distractors present (F_1,9_ = 223.50, p < 0.0001) but, unlike Experiment 1a, the interaction was significant (F_1,9_ = 13.84, p = 0.0002). Splitting the data by distractor number, for 5 distractors the effect of target-pattern combination was significant (F_1,9_ = 11.25, p = 0.0007). Patterned targets on similarly patterned backgrounds produced longer response times than patterned targets on plain backgrounds (mean difference 0.14 s; p = 0.0013) or plain targets on patterned backgrounds (mean difference 0.13 s; p = 0.0022). Plain on patterned and patterned on plain were, however, similar (mean difference 0.01 s; p = 0.6719). For 10 distractors, pattern also had an effect (F_2,18_ = 22.03, p < 0.0001) with patterned on patterned having longer RTs than patterned on plain (mean difference 0.22 s; p = 0.0001) or plain on patterned (mean difference 0.32 s; p < 0.0001), with patterned on plain also having longer RTs than plain on patterned (mean difference 0.11 s; p = 0.0392).

**References**

Hall JR, Cuthill IC, Baddeley R, Shohet AJ, Scott-Samuel NE. 2013. Camouflage, detection and identification of moving targets. Proc R Soc Lond B. 280:20130064.

Yearsley J. 2004. Generate AR1 spatial data. Retrieved from http://www.mathworks.com/matlabcentral/fileexchange/5099-generate-ar1-spatial-data
